# Role of Acetaldehyde in Ethanol Reversal of Tolerance to Morphine-Induced Respiratory Depression in Mice

**DOI:** 10.3389/adar.2021.10143

**Published:** 2022-01-31

**Authors:** Rob Hill, Alexandra Conibear, William Dewey, Eamonn Kelly, Graeme Henderson

**Affiliations:** ^1^ School of Physiology, Pharmacology and Neuroscience, University of Bristol, Bristol, United Kingdom; ^2^ Department of Pharmacology and Toxicology, Virginia Commonwealth University, Richmond, VA, United States

**Keywords:** opioid, ethanol, acetaldehyde, respiration, morphine, tolerance, polydrug, polypharmacology

## Abstract

**Background:** Opioid users regularly consume other drugs such as alcohol (ethanol). Acute administration of ethanol rapidly reverses tolerance to morphine-induced respiratory depression. However, recent research has suggested that the primary metabolite of ethanol, acetaldehyde, may play a key role in mediating the CNS effects seen after ethanol consumption. This research investigated the role of acetaldehyde in ethanol reversal of tolerance to morphine-induced respiratory depression.

**Methods:** Tolerance was induced in mice by 6-days implantation of a 75 mg morphine pellet with control mice implanted with a placebo pellet. Tolerance was assessed by acute morphine administration on day 6 and respiration measured by plethysmography. Levels of acetaldehyde were inhibited or enhanced by pre-treatments with the acetaldehyde chelator D-penicillamine and the inhibitor of acetaldehyde dehydrogenase disulfiram respectively.

**Results:** Morphine pellet implanted mice displayed tolerance to an acute dose of morphine compared to placebo pellet implanted controls. Acute acetaldehyde administration dose-dependently reversed tolerance to morphine respiratory depression. As previously demonstrated, ethanol reversed morphine tolerance, and this was inhibited by D-penicillamine pre-treatment. An acute, low dose of ethanol that did not significantly reverse morphine tolerance was able to do so following disulfiram pre-treatment.

**Conclusion:** These data suggest that acetaldehyde, the primary metabolite of ethanol, is responsible for the reversal of morphine tolerance observed following ethanol administration.

## Introduction

Heroin users are notorious poly drug users, often taking other drugs such as alcohol (ethanol), benzodiazepines, cocaine, gabapentinoids, other illicit drugs as well as other opioids (1–7). Whilst it is known that opioids such as heroin induce fatal overdose by depressing respiration (8, 9), it is important to understand how the contemporaneous use of other drugs may increase the risk of an overdose event.

As ethanol is a central nervous system (CNS) depressant, the general assumption has been that the depressant effects of ethanol and heroin are simply additive, increasing the degree of respiratory depression and thereby increasing the likelihood of an overdose. In a study of overdose deaths involving both ethanol and heroin (10) reported that high blood ethanol levels were associated with intermittent heroin use and this could reflect additivity. However, they also reported a number of deaths in which blood ethanol and morphine were not high (ethanol < 1,000 mg/L and morphine < 0.5 mg/L). Others have reported that blood alcohol levels in fatal heroin overdoses are not typically high (1, 11–14), suggesting another interaction between heroin and ethanol, beyond simple additivity, may also be occurring (15).

We have previously reported that in mice a low dose of ethanol, which by itself did not depress respiration, rapidly reversed tolerance to both morphine- and oxycodone-induced respiratory depression as well as tolerance to their antinociceptive effects (16–20) but did not reverse tolerance induced by methadone (17). We also observed that a low concentration of ethanol (20 mM) could reverse μ opioid receptor (MOR) desensitization and cellular tolerance to morphine in locus coeruleus (LC) neurons as well as tolerance to oxycodone in dorsal root ganglion neurons (20, 21). Ethanol also reduced the phosphorylation of MOR induced by morphine (21). These data suggest that the effect of ethanol is at the level of cells expressing MOR, most likely by reversing receptor desensitization and thus reducing cellular and *in vivo* tolerance to morphine.

There is substantial evidence that MOR desensitization and tolerance to morphine are mediated primarily by a PKC‐dependent mechanism. Exposure to PKC inhibitors reversed morphine-induced MOR desensitization in LC neurons from wild type mice (22, 23) whereas MOR desensitization by morphine was absent in LC neurons from PKCα knock out mice (23). Furthermore, *in vivo* administration of the PKC inhibitors, calphostin C and tamoxifen, significantly reversed morphine- and oxycodone-induced tolerance to respiratory depression (19, 24).

Interestingly, co-administration of a PKC inhibitor and ethanol did not result in enhanced reversal of oxycodone tolerance over ethanol alone (19) suggesting that ethanol may act by inhibiting PKC, thereby reversing morphine tolerance. However, as we have discussed previously (21) the reported effects of ethanol on PKC activity are confusing and contradictory with no effect, slight inhibition and activation having been reported (21, 25–27). This made us consider the possibility that in the brain, acetaldehyde, the primary metabolite of ethanol, rather than ethanol itself might inhibit PKC as has been observed in rat hepatocytes (28) and thus through this mechanism may also reverse morphine tolerance as observed previously through administration of PKC inhibitors.

In the present paper, we have characterised the ability of acetaldehyde to reverse tolerance to morphine respiratory depression and have examined the role of acetaldehyde in ethanol reversal of morphine tolerance.

## Methods and Materials

### Animals

Male CD-1 mice (Charles River, United Kingdom) weighing approximately 30 g were maintained at 22°C on a reversed 12 h dark-light cycle with food and water available ad libitum. All experiments were performed in the dark (active) phase. Mice were randomly ascribed to treatment groups with the experimenter blinded to the drug treatment. All procedures were performed in accordance with the United Kingdom Animals (Scientific Procedures) Act 1986, the European Communities Council Directive (2010/63/EU) and the University of Bristol ethical review document.

### Measurement of Respiration

Respiration was measured in freely moving mice using plethysmography chambers (EMKA Technologies, France) supplied with a 5% CO_2_ in air mixture (BOC Gas Supplies, United Kingdom) as described previously (17). Rate and depth of respiration were recorded and averaged over 5 min periods (except immediately after drug injection when the time period was approximately 3 min) and converted to minute volume (rate × tidal volume).

### Prolonged Morphine Treatment and Assessment of Tolerance

To induce tolerance to morphine mice were implanted subcutaneously for 6 days with a 75 mg morphine pellet on the lower dorsal flank under isoflurane general anaesthesia as described previously (17). Control mice were implanted with placebo pellets. On day 6 following pellet implantation baseline respiration was recorded for 20 min prior to administration of an acute challenge dose of morphine (10 mg/kg i.p.) or vehicle (saline) on one side of the peritoneal cavity. This tolerance protocol was utilised as it has been shown to be sufficient to induce significant tolerance to morphine respiratory depression in mice compared to regular but intermittent administration of morphine (17, 29).

In those experiments in which the effects of acetaldehyde or ethanol on morphine tolerance were studied an i.p. injection of acetaldehyde (50 or 100 mg/kg) or ethanol (0.1 or 0.3 g/kg) or vehicle was administered to the opposite side of the peritoneal cavity immediately after the morphine injection. Respiration was then recorded for 35 min following drug/vehicle administration, allowing maximal depression of respiration to develop. In experiments where disulfiram (40 mg/kg) (30, 31) or D-penicillamine (50 mg/kg) (32) were studied, these were administered i.p. to mice 30 min prior to the administration of morphine and ethanol/acetaldehyde (i.e., 10 min prior to commencing the measurement of respiration). These drugs were administered i.p. on the same side as morphine.

Previously published research administering doses of disulfiram (30 mg/kg) have been shown to significantly inhibit ethanol but not acetaldehyde induced anxiolysis (32), whereas previously published research administering D-penicillamine (50 mg/kg) has shown a clear inhibition of voluntary ethanol consumption (32) with both papers suggesting the effects are mediated through manipulation of acetaldehyde concentrations. These papers were used to define the doses of disulfiram and D-penicillamine used in the current study.

### Data Analysis

For each mouse the change in minute volume, following acute drug administration, has been calculated as a percentage of the pre-drug baseline as described previously (19, 29). Area under the response versus time curve (AUC) was determined using a 100% baseline as described previously (17). Overall changes from a single factor were analysed using a One-way ANOVA with Bonferroni’s post-test. GraphPad Prism 8 was used for all statistical analyses. All data are displayed as mean ± standard error of the mean (s.e.m.).

### Drugs and Chemicals

Morphine alkaloid pellets and placebo pellets were obtained from the National Institute on Drug Abuse (Bethesda, MD, United States). Morphine hydrochloride, (Macfarlan Smith, United Kingdom), D-penicillamine, disulfiram (both from Tocris United Kingdom) were all dissolved as appropriate in sterile saline. Acetaldehyde (Sigma Aldrich United Kingdom), was diluted (w/v) into sterile saline, using only glassware.

## Results

### Induction of Morphine Tolerance

**FIGURE 1:**
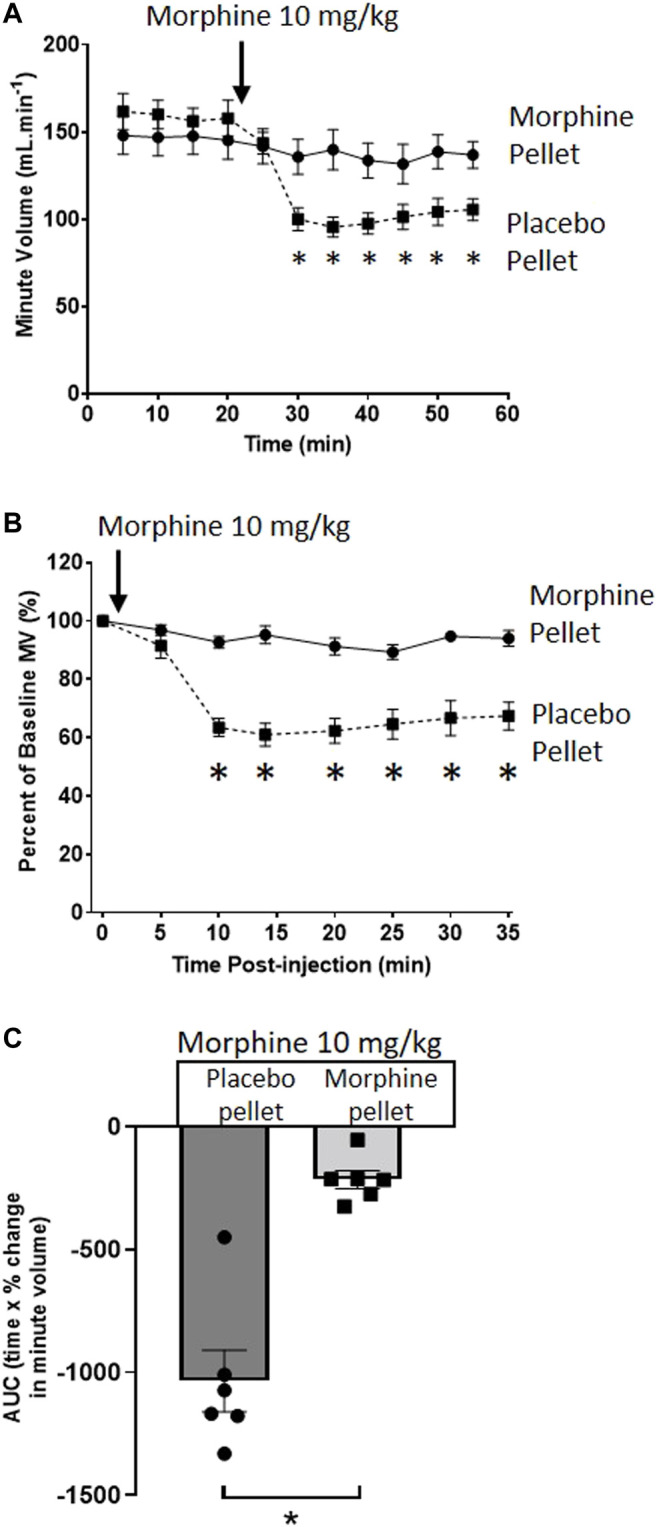
Induction of morphine tolerance. **(A)** Acute injection of morphine (10 mg/kg i.p.), significantly depressed minute volume (MV) in 6 days PP- but not 6 days 75 mg MP-implanted, mice. **(B)** Data in **(A)** replotted as percentage of baseline MV. **(C)** Area under the curve (AUC) calculated from **(B)** (as difference from 100%). All data are presented as mean ± s.e.m. Statistical comparison made by 2-way ANOVA with Bonferroni’s comparison **(A)** (F (DFn, DFd—F (50, 300) = 5.034), **(B)** (F (DFn, DFd—F (7, 70) = 11.96) or unpaired *t*-test **(C)**. * indicates *p* < 0.05 compared to placebo. *n* = 6 for each group.

We have previously described the induction of tolerance to morphine respiratory depression by subcutaneous implantation of a 75 mg morphine pellet (MP) for 6 days (16). Figure 1 illustrates the degree of respiratory depression induced by an acute injection of morphine (10 mg/kg i.p.) in both morphine and placebo pellet (PP) implanted mice. Whilst acute morphine depressed respiration in placebo pellet implanted mice, in morphine pellet implanted mice there was little or no depression of respiration indicating the development of tolerance to the respiratory depressant effect of morphine. Morphine depression of respiration was observed to be primarily mediated by a reduction in the frequency of respiratory events (Table 1), i.e. a decrease in breaths per minute. This is in concurrence with our previously published observations (17) and was consistent throughout all experiments.

**TABLE 1:** Effect of drug treatments on respiratory frequency and tidal volume.

	Pre-drug baseline		15 min drug effect		
Drug treatment	Frequency (BPM)	Tidal volume (ml/breath)	Frequency (BPM)	Tidal volume (ml/breath)	N
Figure 1					
PP + morphine 10 mg/kg	473.2 ± 22.8	0.33 ± 0.02	278.6 ± 17.2^a^	0.35 ± 0.03	6
MP + morphine 10 mg/kg	509.2 ± 36.4	0.29 ± 0.02	477.4 ± 35.7	0.28 ± 0.01	6
Figure 2					
MP + acet. 50 mg/kg	460.1 ± 31.4	0.33 ± 0.02	507.1 ± 16.1	0.29 ± 0.04	6
MP + acet. 100 mg/kg	529.0 ± 44.8	0.27 ± 0.02	494.6 ± 54.1	0.28 ± 0.02	6
MP + acet. 50 mg/kg and morphine 10 mg/kg	348.1 ± 12.2	0.43 ± 0.04	286.4 ± 13.1^a^	0.44 ± 0.02	6
MP + acet. 100 mg/kg and morphine 10 mg/kg	427.6 ± 16.0	0.32 ± 0.01	310.8 ± 34.7^a^	0.31 ± 0.02	6
Figure 3					
PP + morphine 10 mg/kg and ethanol 0.3 g/kg (Veh pre-treatment)	409.9 ± 11.8	0.35 ± 0.02	226.0 ± 18.8^a^	0.37 ± 0.01	6
PP + morphine 10 mg/kg and ethanol 0.3 g/kg (DP 50 mg/kg pre-treatment)	438.1 ± 20.1	0.36 ± 0.02	259.3 ± 31.5^a^	0.39 ± 0.03	6
MP + saline (DP 50 mg/kg pre-treatment)	415.7 ± 15.7	0.36 ± 0.02	465.9 ± 19.4	0.31 ± 0.02	6
MP + morphine 10 mg/kg (DP 50 mg/kg pre-treatment)	448.3 ± 20.9	0.32 ± 0.03	445.9 ± 23.1	0.31 ± 0.02	6
MP + ethanol 0.3 g/kg (DP pre-treatment)	501.7 ± 36.4	0.30 ± 0.02	509.2 ± 36.4	0.33 ± 0.01	6
MP + morphine 10 mg/kg (veh pre-treatment)	454.6 ± 32.9	0.33 ± 0.02	407.4 ± 26.9	0.34 ± 0.01	6
MP + morphine 10 mg/kg and ethanol 0.3 g/kg (veh pre-treatment)	424.8 ± 19.6	0.37 ± 0.02	226.7 ± 20.4^a^	0.35 ± 0.01	6
MP + morphine 10 mg/kg and ethanol 0.3 g/kg (DP 50 mg/kg pre-treatment)	327.7 ± 12.3	0.40 ± 0.03	261.0 ± 9.2^a^	0.44 ± 0.01	6
Figure 4					
MP + morphine 10 mg/kg (veh pre-treatment)	522.4 ± 18.1	0.29 ± 0.02	461.8 ± 18.6	0.31 ± 0.02	6
MP + morphine 10 mg/kg and ethanol 0.1 g/kg (veh pre-treatment)	425.8 ± 22.2	0.37 ± 0.01	277.3 ± 18.2^a^	0.44 ± 0.02^a^	6
MP + morphine 10 mg/kg (disulfiram 40 mg/kg pre-treatment)	471.9 ± 42.5	0.32 ± 0.02	401.9 ± 45.4	0.36 ± 0.03	6
MP + morphine 10 mg/kg and ethanol 0.3 g/kg (disulfiram 40 mg/kg pre-treatment)	485.6 ± 25.1	0.30 ± 0.02	296.3 ± 21.4*	0.33 ± 0.01	6

a Indicates a significant change (*p* < 0.05) from pre-drug baseline values.

All values are mean ± SEM, of 5-min averages. Pre-drug baseline values are taken from the 15–20-min pre-drug time bin. Post-drug values are taken from the 15–20-min time bin taken from the time of injection. Unless otherwise stated there was no significant change from pre-drug baseline levels. MP, 75-mg morphine pellet, acet, acetaldehyde; DP, D-penicillamine. Values were compared using a paired two-way Student’s *t*-test.

### Reversal of Morphine Tolerance by Acute Acetaldehyde Administration

**FIGURE 2:**
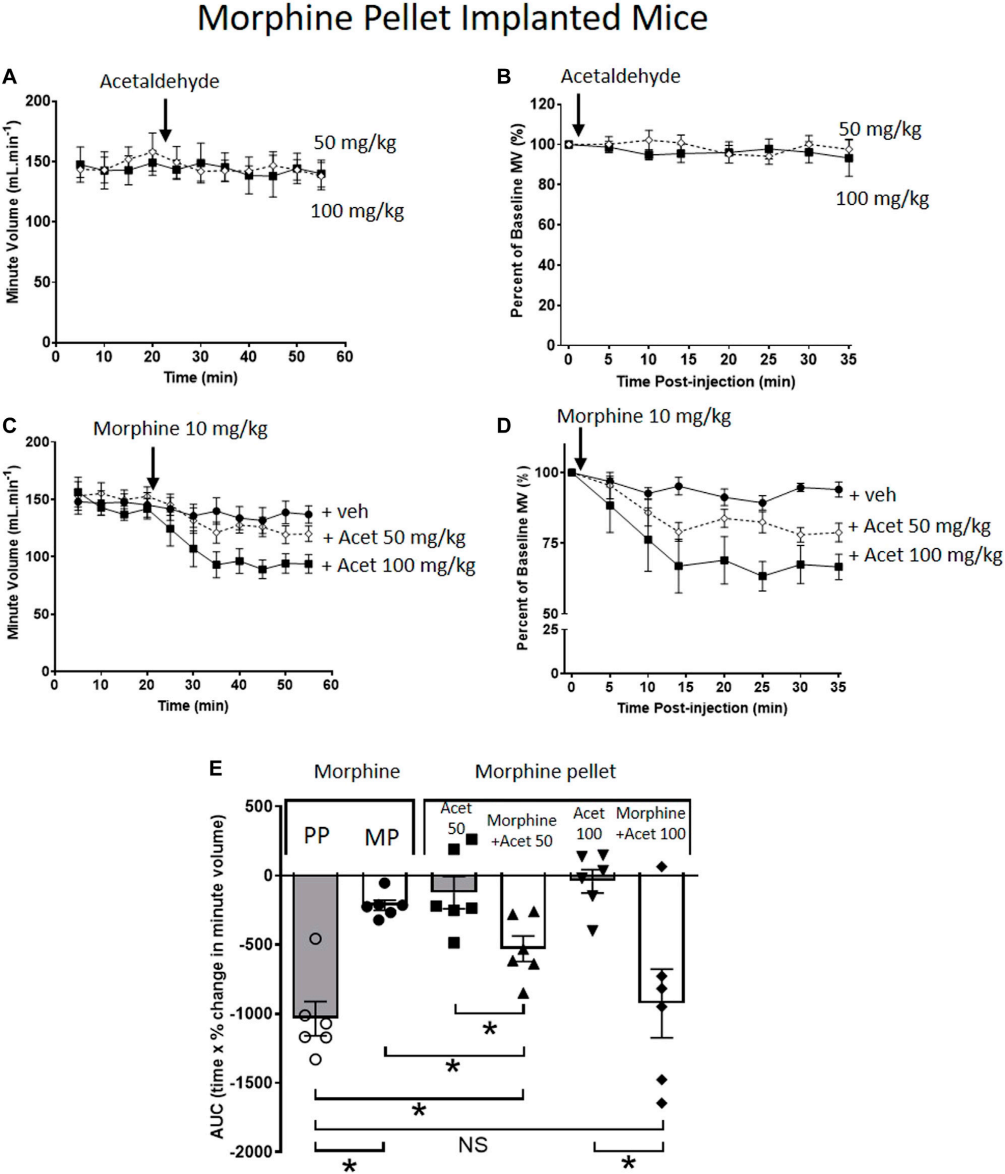
Acetaldehyde modulates morphine tolerance. **(A,B)** Acute administration of acetaldehyde 50 mg/kg i.p (open symbols) or 100 mg/kg i.p. (closed symbols) did not depress minute volume (MV) in mice implanted for 6 days with a 75 mg MP. **(C,D)** Acute administration of morphine (10 mg/kg i.p.) did not depress respiration in MP- implanted mice indicating that tolerance had developed. Co-administration of acetaldehyde (Acet) and morphine depressed respiration in an acetaldehyde dose-dependent manner. **(E)** Area under the curve (AUC) analysis of data in **(B,D)** shows that when acetaldehyde 50 mg/kg or 100 mg/kg was administered along with acute morphine respiratory depression was restored i.e. morphine tolerance had been reversed. MP, morphine pellet; PP, placebo pellet. All data are presented as mean ± s.e.m. Statistical comparison made by One-way ANOVA with Bonferroni’s comparison (F (DFn, DFd—F (5, 29) = 9.971) in **(E)**. * indicates *p* < 0.05 as shown. *n* = 6 for each group.

When administered alone to MP-implanted mice, acetaldehyde (50 and 100 mg/kg i.p.) did not alter respiration (Figures 2A,B,E). In contrast, co-administration of acetaldehyde with an acute dose of morphine (10 mg/kg i.p.) in MP-implanted mice, dose-dependently increased the degree of respiratory depression seen when compared to respiratory depression induced by the same acute dose of morphine co-administered with vehicle (Figures 2C–E). Area under the curve (AUC) analysis shows that the degree of respiratory depression induced by morphine co-administered with acetaldehyde 100 mg/kg in MP-implanted mice was not significantly different from that seen when morphine alone was administered to PP-implanted mice (Figure 2E) demonstrating that tolerance to morphine induced by morphine pellet implantation had been completely reversed.

### Effect of Changes in Acetaldehyde Levels on Ethanol Reversal of Morphine Tolerance

To investigate whether acetaldehyde plays a role in ethanol reversal of morphine tolerance, we used two agents that would alter the levels of acetaldehyde in the brain following ethanol administration. D-penicillamine chelates acetaldehyde without affecting ethanol metabolism, thereby reducing free acetaldehyde levels (32–34). Whereas, disulfiram inhibits the metabolism of acetaldehyde by acetaldehyde dehydrogenase, thereby increasing and prolonging acetaldehyde levels in the brain following ethanol administration (35–37).

**FIGURE 3:**
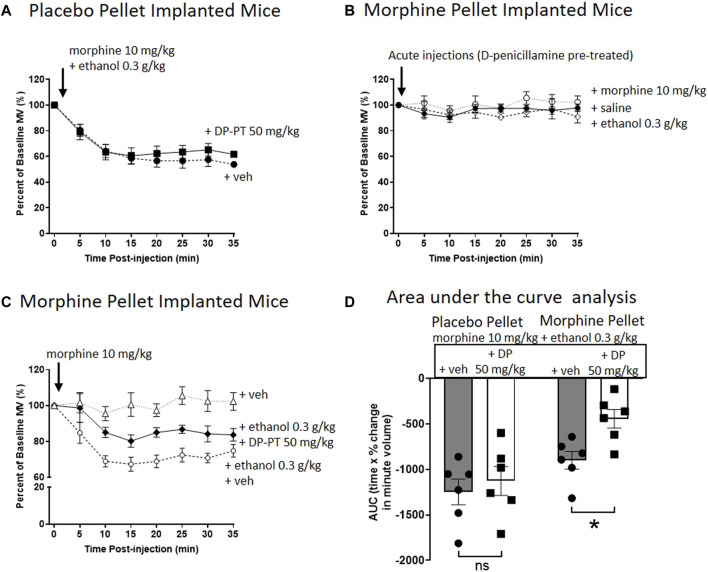
Pre-treatment with D-penicillamine inhibits ethanol reversal of morphine tolerance. **(A)** Morphine (10 mg/kg i.p.) co-administered with ethanol (0.3 g/kg i.p.) to 6 days placebo pellet implanted mice induced the same level of respiratory depression with and without a 30 min pre-treatment (PT) with D-penicillamine (DP, 50 mg/kg i.p.). **(B)** Administration of morphine, ethanol or saline (i.p.) to 6 days 75 mg morphine pellet implanted mice did not depress respiration following a 30 min pre-treatment with D-penicillamine. **(C)** Pre-treatment with D-penicillamine (DP, 50 mg/kg i.p.) significantly reduced the level of respiratory depression induced by morphine and ethanol co-administration in 6 days MP- implanted mice. **(D)** Area under the curve (AUC) analysis of data in **(A,C)**. MP, morphine pellet; PP, placebo pellet. All data are presented as mean ± s.e.m. Statistical comparison made by Two-way ANOVA with Bonferroni’s comparison (F (DFn, DFd—F (1, 20) = 5.094) in **(D)**. * indicates *p* < 0.05 as shown. *n* = 6 for each group.

In PP-implanted mice, pre-treatment for 30 min with D-penicillamine (50 mg/kg i.p.) did not alter the degree of respiratory depression induced by co-administration of morphine (10 mg/kg i.p.) and ethanol (0.3 g/kg i.p.) (Figure 3A). In MP-implanted mice, acute administration of saline, morphine or ethanol did not result in respiratory depression following pre-treatment with D-penicillamine (Figure 3B). Co-administration of ethanol and morphine in saline pre-treated, MP-implanted mice resulted in significant respiratory depression compared to morphine co-administered with saline (Figures 3C,D). This demonstrates ethanol reversal of morphine tolerance as previously described (16, 17). In contrast however, pre-treatment with D-penicillamine prior to acute morphine and ethanol administration resulted in a significant reduction in the respiratory depression seen with morphine and ethanol in MP-implanted mice (Figures 3C,D).

**FIGURE 4:**
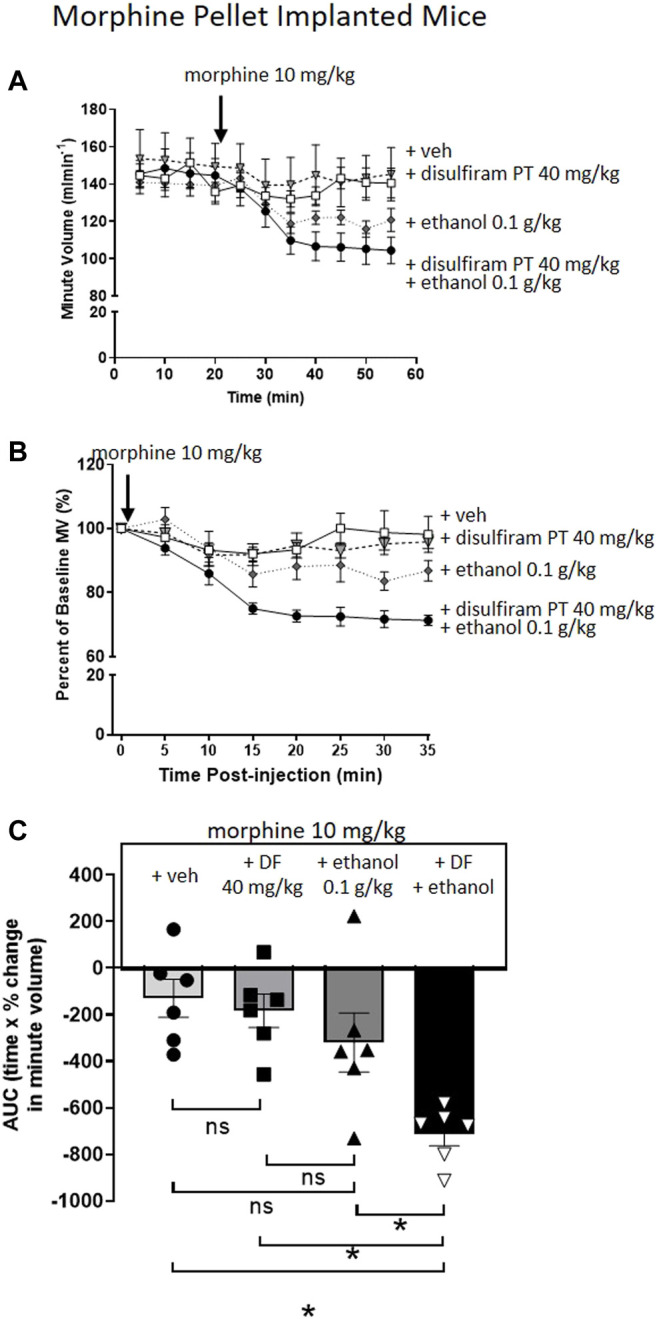
Pre-treatment with disulfiram enhances ethanol reversal of morphine tolerance. **(A,B)** Morphine (10 mg/kg i.p.) induced no respiratory depression in 6 days 75 mg MP-implanted mice with or without a 30 min disulfiram (DF) (40 mg/kg i.p.) pre-treatment. Similarly, co-administration of morphine with a low dose of ethanol (0.1 g/kg i.p.) did not induce significant respiratory depression in MP- implanted mice. Pre-treatment (PT) with disulfiram (40 mg/kg i.p.) in morphine pellet implanted mice enhanced the degree of respiratory depression to co-administration of morphine and ethanol. **(C)** Area under the curve (AUC) analysis of data in **(B)** shows that pre-treatment with disulfiram (40 mg/kg i.p.) in MP- implanted mice prior to acute co-administration of morphine and ethanol significantly enhanced the degree of respiratory depression observed when compared to morphine and ethanol co-administered in vehicle pre-treated mice. MP, morphine pellet; PP, placebo pellet. All data are presented as mean ± s.e.m. Statistical comparison made by One-way ANOVA **(C)** with Bonferroni’s comparison (F (DFn, DFd—F (3, 20) = 0.570). * indicates *p* < 0.05 as shown. *n* = 6 for each group.

To investigate the effect of disulfiram, which would be expected to enhance acetaldehyde levels, we chose to examine its ability to enhance the reversal of morphine tolerance by a low dose of ethanol (0.1 g/kg i.p.) that alone produced no significant change in morphine tolerance (Figures 4A–C). Pre-treatment for 30 min with disulfiram (40 mg/kg i.p.) or saline in MP-implanted mice did not alter the tolerance observed following acute morphine (10 mg/kg i.p.) administration (Figures 4A–C). However, pre-treatment with disulfiram significantly enhanced the degree of respiratory depression observed following morphine and low dose ethanol administration to MP-implanted mice (Figures 4A–C). Taken together our findings with D-penicillamine and disulfiram suggest that acetaldehyde, as a metabolite of ethanol, is responsible for the reversal of morphine tolerance by ethanol.

## Discussion

In the present paper, we have provided evidence that acetaldehyde is the mediator of ethanol reversal of morphine tolerance to respiratory depression. We first demonstrated that acute administration of acetaldehyde itself reversed tolerance to morphine-induced respiratory depression in a manner similar to ethanol. Acetaldehyde can be produced from ethanol in the brain through metabolism by brain catalase and CYP2E1 enzymes and has been suggested to mediate many of the psychopharmacological effects of ethanol (38, 39). Acetaldehyde levels in the brain can be manipulated in two ways, chelation by D-penicillamine to reduce free acetaldehyde levels (32–34) and inhibition of acetaldehyde metabolism by disulfiram, an acetaldehyde dehydrogenase inhibitor, to elevate acetaldehyde levels (35–37). We observed that chelation of acetaldehyde with D-penicillamine reduced the reversal of tolerance by ethanol and that disulfiram, which inhibits acetaldehyde metabolism, potentiated the reversal of tolerance by ethanol. These data strongly support the view that acetaldehyde plays a significant role as a mediator of ethanol reversal of morphine tolerance to respiratory depression.

Disulfiram is used in the treatment of recovering alcoholics (34, 40, 41). In humans taking disulfiram subsequent to consumption of alcohol results in a peripheral accumulation of acetaldehyde that evokes a highly aversive response and is intended to act as a deterrent therapy to prevent relapse (41, 42), though the efficacy of this has been questioned (40–42). In mice administered acetaldehyde or pre-treated with disulfiram and then administered ethanol we did not observe any changes in normal spontaneous behaviours (e.g., excessive grooming of the injection site, writhing, squirming, extreme quiescence) that might indicate an aversive response although we did not specifically test for aversion in behavioural studies. It has previously been reported that acetaldehyde (100 mg/kg) induced a small but significant degree of anxiogenesis in mice tested in the elevated plus maze (31), even though this dose of acetaldehyde did not result in significantly increased levels of the stress hormone corticosterone. The data in our experiments supports this as there did not appear to be any significant degree of anxiety produced by acetaldehyde that might have affected the breathing of the mice.

Agonist induced MOR desensitisation is thought to be an important component of opioid tolerance (43). For morphine, a relatively low efficacy MOR agonist (44), there is good evidence that PKC is one of the major mediators of MOR desensitization and tolerance (43), though both G-protein receptor kinases and arrestin are also thought to have lesser roles in mediating morphine induced MOR desensitisation (45–47). We and others have previously demonstrated that PKC is an important mediator of cellular tolerance (22) as well as tolerance to its antinociceptive effects (48–50). We have also demonstrated that the PKC inhibitors calphostin C and tamoxifen, were able to reverse tolerance to the respiratory depressant effects of morphine (23). PKCα, PKCγ and to a lesser extent PKCε have been implicated in mediating morphine antinociceptive tolerance (22, 50). In locus coeruleus neurons PKCα is the isoform involved in morphine-induced MOPr desensitization (23). We have recently observed an inability of morphine to induce tolerance to respiratory depression in PKCα knock out mice (Hill and Henderson, unpublished observation). Morphine tolerance can be enhanced by expressing a constitutively active form of PKCα in respiratory control neurons in mice (51). These data suggest a key role for PKCα in tolerance to morphine respiratory depression.

We had previously suggested that ethanol might reverse morphine tolerance through inhibition of PKCα (21) although using purified PKCα we were only able to demonstrate slight (∼20%) inhibition of enzyme activity *in vitro* at a relatively high concentration (100 mM) of ethanol. Other investigators had reported conflicting effects of ethanol on PKC activity, some observing no effect, slight inhibition or even activation (25–27). Importantly, acetaldehyde has been shown to inhibit PKC activity in rat hepatocytes (28) and it was this activity in conjunction with the previously observed ability of ethanol and PKC inhibition to independently and non-summatively reverse morphine tolerance, that suggested acetaldehyde may be playing a key role at the level of PKC in this process. In recent years use of gabapentinoids, gabapentin and pregabalin, amongst people with a history of opioid use has increased (4, 6, 52–55) and may contribute to overdose deaths (56, 57). We found that, like ethanol, pregabalin reversed tolerance to respiratory depression induced by morphine (4, 19). Gabapentinoids are known to interfere with the translocation of the α2δ1 calcium channel subunit to the plasma membrane (58). It will be of interest to determine whether these drugs can also interact with PKC activation in vitro assays.

We have also previously shown that prolonged ethanol administration to mice through a liquid ethanol diet was able to prevent the development of tolerance to morphine respiratory depression (59). These data in conjunction with the currently presented research suggest that prolonged ethanol consumption in heroin users may present significant levels of centrally formed acetaldehyde that chronically inhibits mechanisms of tolerance at the level of the MOR. Whilst the manner of tolerance induction in the presented research is not intended to mimic the opioid users consumption patterns, these data suggest that chronic or acute consumption of ethanol is likely to increase the risk of accidental overdose, particularly in more experienced users where established tolerance may be reversed by ethanol consumption (15).

Further research investigating the effect of centrally administered acetaldehyde on the development and reversal of tolerance to morphine respiratory depression as well as the role of acetaldehyde in the effects of prolonged ethanol administration will be important future avenues of research to clarify further the role of acetaldehyde in ethanol reversal of tolerance to morphine. Importantly, further research should focus on establishing a mechanistic action through which PKC activity or location is altered by ethanol, acetaldehyde and pregabalin. This is likely to provide important insight not only into potentially dangerous polypharmacological scenarios, but also mechanisms through which patient analgesia may be enhanced in incidences of tolerance. The relative risk that this presents with regards to enhanced respiratory depression will need to be carefully considered in this context.

## Conclusion

We propose that ethanol reversal of morphine tolerance to respiratory depression results from its conversion to acetaldehyde and that it is acetaldehyde, rather than ethanol, which fundamentally interacts with mechanisms of tolerance effective at the MOR.

## Data Availability

The raw data supporting the conclusion of this article will be made available by the authors, without undue reservation.
